# The Role of Radiation in Cancer Treatment: New Insights towards Personalized Therapies

**DOI:** 10.3390/jpm12020312

**Published:** 2022-02-19

**Authors:** Luigi Minafra, Francesco P. Cammarata, Marco Calvaruso

**Affiliations:** Institute of Bioimaging and Molecular Physiology-National Research Council (IBFM-CNR), 90015 Cefalù, Italy; francesco.cammarata@ibfm.cnr.it (F.P.C.); marco.calvaruso@ibfm.cnr.it (M.C.)

Despite all the recent pharmacological advances and the introduction of targeted therapies in clinical practice, cancer still remains one of the leading cause of death, accounting for 10 million deaths per year, based on the most recent reports [1]. Cancer treatment relies on three main pillars, represented by: surgery, chemotherapy, and radiotherapy (RT). In recent years, tailored therapies have mainly involved the development of pharmacological approaches to increase the efficacy of drugs and to broaden the horizons of precision medicine. The current use of monoclonal antibodies and kinase inhibitors in tumor management, are key steps in this context [2]. Nowadays, patients are stratified not only considering the histological nature of the type of cancer which affects them, but also the presence of distinctive biomarkers to improve the efficacy of their therapies. Although the idea of targeted therapies has permitted overcoming the “one-size-fits-all” hypothesis for chemotherapy, this assumption still remains for RT [3].

RT represents a fundamental component of multimodality approaches for the treatment of many types of cancer. Approximately, the 50–60% of all cancer patients are undergoing RT through several modalities, such as internal or external RT, either alone or in combination with other treatment regimens (i.e., surgery or chemotherapy). Improvements in RT have been made in regard to the reach of higher technological levels, which allow an optimal dose distribution and conformational treatment customization. Research in both radiobiology and medical physics is focused on the evaluation of new hypofractionated or combined RT protocols, in order to increase effectiveness in cohorts of patients [4]. However, to date, the overall dose delivered is the same for patients with the same organ tumor, despite a strong heterogeneity among tumor subtypes [5].

Indeed, tumors can be classified as radioresistant and radiosensitive and such balance is not only dependent on the nature of the radiation used to treat the tumor bulk, but also by different complex factors, increasingly highlighted by radiobiology studies [6]. They include the genetic background of the patient and all those other cellular and molecular factors that orchestrate the different response of the tumor and healthy tissues to RT, such as, activation of DNA repair pathways, differential gene expression profiling, tumor stem cells, hypoxia, and tumor microenviroment [7,8].

In this scenario, the role of cancer research is to identify specific molecular traits of neoplasms, in order to design successful clinical treatments. Therefore, in the era of target therapies, the identification of cancer biomarkers is required to drive therapeutic decisions also between different RT modalities and schedules. The use of omics sciences provides new high throughput tools to monitor tumor progression and to predict radiation response both in tumor and healthy tissue [9,10,11].

This Special Issue aims to describe the state of the art about the role of RT in cancer and its contribution for personalized medicine.

More specifically, Musielak and colleagues collected in a review paper the advancement in regard to the use of proton therapy (PT) in minimizing the toxicity of breast cancer (BC) RT. As is already known, PT is considered a valuable alternative to conventional RT modalities for its ballistic precision and capability to spare the healthy tissue surrounding the tumor bulk. When considering BC, one of the main side effects of RT is cardiotoxicity. Hence, the preservation of cardiac tissue represents a main challenge and authors described a thorough analysis of literature regarding issues related to RT-induced cardiac toxicity [12].

In line with this topic, Cunningham et al. studied the impact of breast size on dosimetry, comparing PT versus conventional X-ray RT for BC, with the use of two different innovative RT techniques, such as hybrid inverse-planned intensity modulated X-ray radiotherapy (h-IMRT) and intensity modulated proton therapy (IMPT) [13].

In order to underline radioresponse biomarkers for glioblastoma multiforme (GBM), Bravatà et al., performed a transcriptomic study by whole-genome cDNA microarray on the U87 GBM cell line following PT treatment in hypoxic conditions [14]. Hypoxia represents one of the main factors inducing radioresistance and poor prognosis for GBM [15]. Therefore, the identification of molecular pathways linked to radioresistance could suggest new molecular targets to develop innovative and more effective targeted drugs as adjuvants in PT.

In order to depict the genetic basis of radiation-induced late skin toxicity, Cargnin et al. carried out a targeted next-generation sequencing study, in breast cancer patients, for the identification of genetic predictors of healthy skin response to RT, as well as cardiotoxicity; in fact, RT induces detrimental side effects to skin tissue [16].

Finally, Danieli et al., provided a thorough description about the state of the art of all those strategies adopted in personalized dosimetry aimed at targeted radiation therapy (TRT) for the treatment of different types of cancer [17].

## Data Availability

Not applicable.
